# Effects of Hydraulic Diameters on CO_2_ Absorption in Flat-Plate Membrane Contactors with Inserted S-Ribbed Carbon Fiber Turbulence Promoters

**DOI:** 10.3390/membranes16050162

**Published:** 2026-04-30

**Authors:** Chii-Dong Ho, Ping-Cheng Hsieh, Thiam Leng Chew, Jyun-Jhe Li

**Affiliations:** 1 Department of Chemical and Materials Engineering, Tamkang University, Tamsui, New Taipei 251301, Taiwan; 2 Department of Chemical Engineering, Faculty of Engineering, Universiti Teknologi Petronas, Seri Iskandar 32610, Perak, Malaysia; thiamleng.chew@utp.edu.my; 3 CO2 Research Center (CO2RES), Institute of Contaminant Management, Universiti Teknologi Petronas, Seri Iskandar 32610, Perak, Malaysia

**Keywords:** S-ribbed carbon fiber promoters, CO_2_ membrane absorption, enhanced Sherwood numbers, flat-plate membrane absorption modules, concentration polarization effect, hydraulic widths

## Abstract

One-dimensional mass transfer resistance-in-series framework was developed theoretically and validated experimentally using a flat-plate polytetrafluoroethylene/polypropylene (PTFE/PP) membrane module to predict CO_2_ absorption fluxes and concentration distributions. The decline in CO_2_ absorption efficiency along the membrane module is primarily attributed to increased concentration polarization resistance and a reduced driving force concentration gradient. To alleviate these limitations, carbon fiber promoters were strategically embedded to suppress concentration polarization, reduce the mass transfer resistances, and enhance turbulence intensity. In the present study, device performance was further improved by implementing properly ascending or descending hydraulic equivalent widths along the absorbent feed channel. Under the descending configuration, an absorption flux enhancement of up to 44.94% was achieved relative to an empty-channel module (i.e., without S-ribbed carbon fiber inserts). Theoretical formulations were established to predict absorption fluxes under varying monoethanolamine (MEA) volumetric flow rates, CO_2_/N_2_ mixture flow rates, and inlet CO_2_ feed concentrations. The model predictions showed good agreement with experimental results obtained using MEA solutions under both ascending and descending hydraulic width operations, demonstrating effective mitigation of polarization effects and enhanced absorption flux along the absorbent feed channel. An economic assessment of the S-ribbed carbon fiber module was conducted by evaluating the trade-off between absorption flux enhancement and incremental power consumption. The results indicate that the proposed design provides a practical and economically viable approach for improving the performance of membrane-based CO_2_ capture technologies. In addition, an enhanced Sherwood number correlation, expressed in a simplified form, was developed and employed to estimate the mass transfer coefficients of CO_2_ membrane absorption modules incorporating S-ribbed carbon fiber promoters.

## 1. Introduction

A membrane contactor module enables effective contact between a liquid absorbent and flue gas by providing an extensive interfacial area for mass transfer, in which soluble gas components are selectively absorbed on the liquid-phase membrane surface in liquid–liquid or gas–liquid systems [1]. Membrane technology has been widely applied for the selective purification of CO_2_ from gas mixtures, achieving high absorption efficiency [2] by facilitating CO_2_ diffusion across hydrophobic membrane systems [3]. This technology is favored for its high efficiency, low power consumption, large mass transfer area, and scalability in continuous operations [4]. Flue gas produced by fossil fuel combustion contains substantial CO_2_, so separation is required to curb climate-related impacts [5]. In biogas upgrading and stabilization, the main contaminants including CO_2_ (30–45%) and H_2_S (0.5–1%) in specification are reduced because both are linked to greenhouse forcing and broader climate change [6,7]. Meeting these quality targets increases the market value of the product gas and enables downstream processing, conditioning, and pipeline delivery. However, a decline in CO_2_ absorption efficiency along the membrane module is primarily attributed to increased concentration polarization resistance and a reduced concentration driving force gradient. A key performance constraint in many membrane operations is concentration polarization, which diminishes the driving force and, therefore, reduces transmembrane flux [8]. Prior work shows that introducing stronger mixing through the use of surface roughness [9], filament-packed passages [10], or carbon fiber slot-channel configurations [11] can weaken polarization by disrupting the near-wall boundary layer. Building on this rationale, carbon fiber spacers were embedded to suppress polarization, lower mass transfer resistance and intensify eddy activity relative to conventional devices. Previous studies have demonstrated that incorporating eddy promoters into membrane contactors effectively reduces concentration polarization resistance by increasing turbulence intensity, thereby improving vapor permeate flux, albeit at the expense of increased power consumption. In the present work, device performance was further enhanced by systematically ascending or descending the widths of S-ribbed carbon fibers along the absorbent feed channel. Under the descending configuration, absorption flux enhancement of up to 44.91% was achieved compared with that of a module employing an empty channel without S-ribbed carbon fiber promoters. To quantify how polarization limits an MD system and to identify designs that scale, membrane effectiveness was evaluated by comparing flux gains against the associated energy penalty. CO_2_ capture under turbulent conditions was examined using a parallel-plate gas/liquid membrane contactor fitted with eddy promoters [12]. By generating vortices and secondary flows, these inserts create wakes that continually renew the boundary layer region and enhance interfacial mass transfer [13]. Because turbulence promoters typically raise pressure drop [14], their use is commonly framed as a trade-off between absorption rate improvement and power consumption when assessing module economics [15]. Consistent with this perspective, the S-ribbed carbon fiber module was assessed by jointly considering absorption flux enhancement and incremental pumping power, alongside evidence that improved hydrodynamics can translate to better performance in modules and commercial-scale pilots/applications [16]. Accordingly, this study proposes S-ribbed carbon fiber promoters with ascending and descending hydraulic width arrangements to reduce mass transfer resistance while keeping the added power demand within a controllable range. Accordingly, the economic feasibility of the S-ribbed carbon fiber module was evaluated by jointly considering absorption flux enhancement and incremental power consumption. In this study, incorporating ascending and descending hydraulic widths of S-ribbed carbon fiber promoters were proposed to effectively reduce undesired mass transfer resistance for economic viability while considering a manageable increase in power consumption.

A one-dimensional model was developed to simulate CO_2_ absorption rates and concentration profiles as functions of MEA liquid flow rate, CO_2_/N_2_ gas flow rate, and inlet CO_2_ concentration. Because the diffusion–reaction process is isothermal and uses MEA as the absorbent, the analysis anticipates rise in overall mass transfer resistance along the module length together with more pronounced concentration polarization, particularly in the downstream section. The formulation combines species mass balances with reaction-kinetic expressions and was validated experimentally using a flat-plate PTFE/PP membrane module. Transport within the membrane was represented using the dusty gas model [17], enabling simultaneous treatment of diffusion pathways and reactive uptake effects [18]. This modeling approach is widely used to evaluate process performance [19] for alkanolamine-based capture systems [20] and to probe the mechanisms governing CO_2_ absorption in such contactors [21]. For industrial deployment, membrane contactors are attractive due to their low solvent vapor pressure, thermal robustness, continuous operation capability, modular design, and scalable configuration [22]. In addition to amine-based absorption, imidazole ionic liquids have drawn substantial interest as alternative absorbents because they can provide higher CO_2_ solubility, faster diffusion, and improved permeability in gas-separation applications across multiple industrial settings [23]. Absorption efficiency was analyzed and correlated using a mass transfer enhancement factor. Accordingly, the effects of S-ribbed carbon fiber promoter widths, MEA feed concentration, and gas and liquid flow rates on CO_2_ absorption flux were systematically evaluated under various configurations. The mass transfer behavior across the membrane was formulated in a manner analogous to that of modules employing uniform S-ribbed carbon fiber widths [24], while extending the analysis to channels with variable S-ribbed carbon fiber widths. Theoretical predictions showed good agreement with experimental results obtained using MEA solutions in both ascending and descending hydraulic width channels, confirming the effectiveness of S-ribbed carbon fiber structures in mitigating concentration polarization and enhancing absorption performance.

To support the design and evaluation of S-ribbed carbon fiber channels in flat-plate membrane contactors, a generalized (and intentionally simplified) Sherwood number correlation was established for predicting the CO_2_ mass transfer coefficient, offering a practical metric for performance screening. The results suggest that tailoring S-ribbed carbon fiber geometries can raise CO_2_ absorption flux and, in turn, improve both device performance and the economic case for membrane-based CO_2_ absorption. Further optimization was explored by introducing S-ribbed carbon fiber turbulence promoters with ascending and descending hydraulic width arrangements while explicitly considering the accompanying energy requirement. Model predictions show that the descending width configuration yields a larger absorption flux gain per unit increase in energy consumption than designs without S-ribbed promoters. The mathematical framework proposed for S-ribbed carbon fiber-filled modules is broadly transferable and can be extended to other hydrophobic membrane systems.

## 2. Theory and Analysis

**Figure 1:**
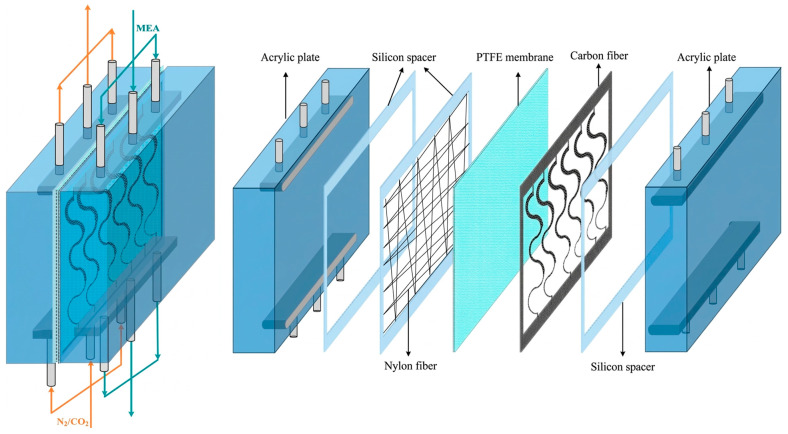
Three regions considered for modeling CO_2_ absorption in a flat-plate gas/liquid membrane contactor.

A depiction of the mass transfer rate in the membrane contactor is represented in Figure 1 for schematic representations of ascending S-ribbed carbon fiber width operations in developing the mathematical modeling of CO_2_ absorption in MEA solution as an absorbent, through which the device performances were obtained to make comparisons with those in the previous work [24] with the uniform S-ribbed carbon fiber widths.

**Figure 2:**
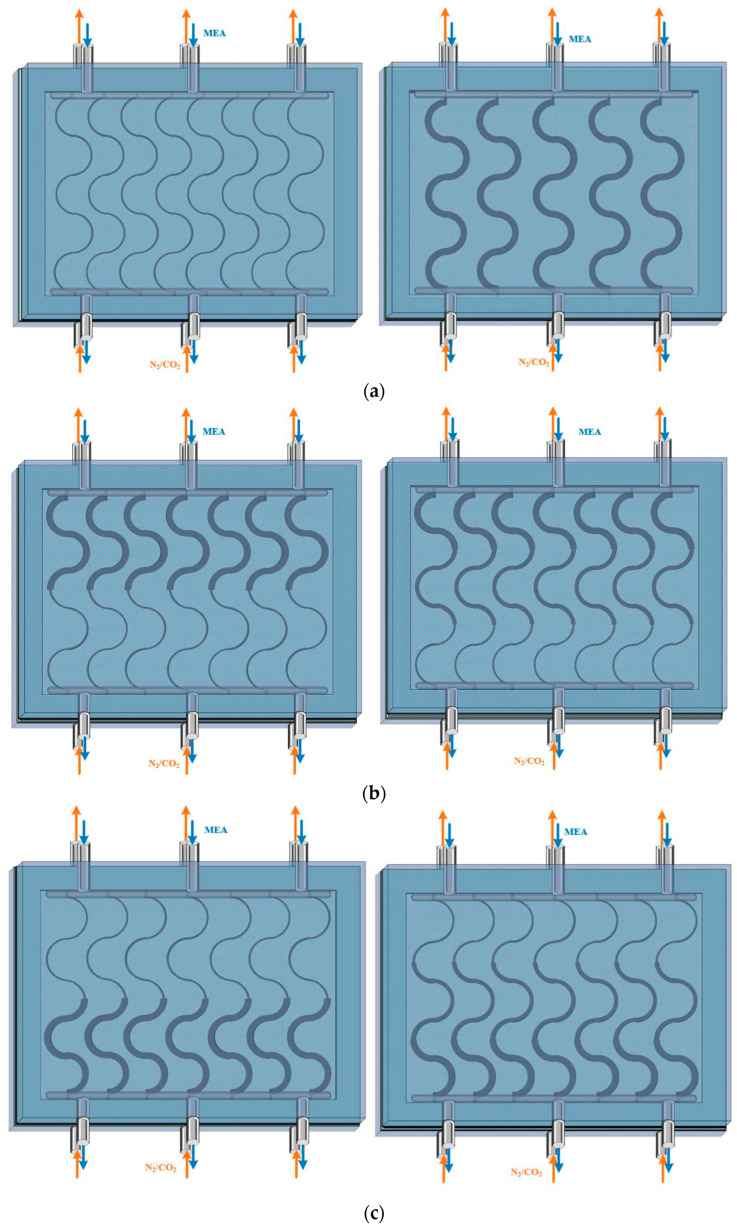
Configurations of module with various S-ribs carbon fiber width filaments inserted. (**a**) Uniform carbon fiber widths; (**b**) Descending carbon fiber widths for MEA absorbent feed stream; (**c**) Ascending carbon fiber widths for MEA absorbent feed stream.

**Figure 3:**
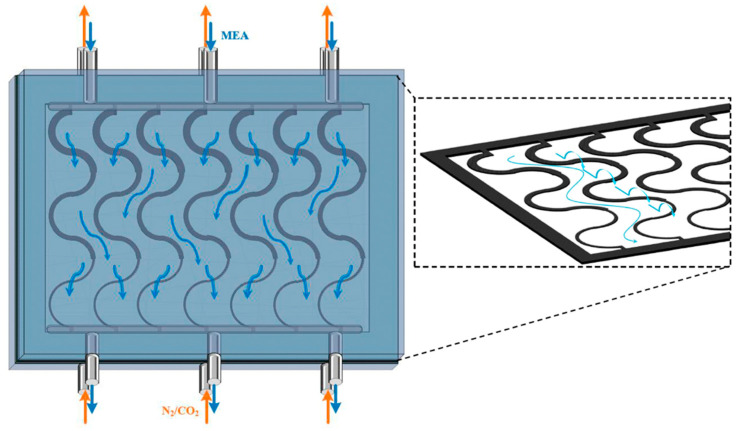
MEA absorbent flow streamlines in the descending S-ribs carbon fiber width channel with hydrodynamic angles under countercurrent-flow operation. (Brown arrow for N_2_/CO_2_ feed stream and blue arrow for MEA feed stream).

Figure 2 presents top-view schematics of three curved S-ribbed carbon fiber turbulence promoter geometries with various hydraulic widths used as design parameters, as illustrated in Figure 2a–c. The schematics depict S-ribbed carbon fiber promoters arranged in both ascending and descending width configurations, covering approximately 13% of the hydrophobic membrane surface. These structures function as eddy promoters to enhance turbulence intensity while simultaneously providing mechanical support to suppress membrane vibration. The investigated hydraulic width configurations include: (a) uniform promoter widths of 3 mm and 5 mm; (b) ascending promoter widths of 3–5 mm and 3–4–5 mm; and (c) descending promoter widths of 5–3 mm and 5–4–3 mm. These promoters were mounted on the membrane surface and served as key design parameters in the present study. Turbulence intensity enhancement was achieved by incorporating S-ribbed carbon fiber turbulence promoters with various array configurations and hydraulic widths into the MEA absorbent feed channel under countercurrent-flow operation. The descending configurations and corresponding hydrodynamic flow angles are illustrated in Figure 3.

### 2.1. Mass Transfer

**Figure 4:**
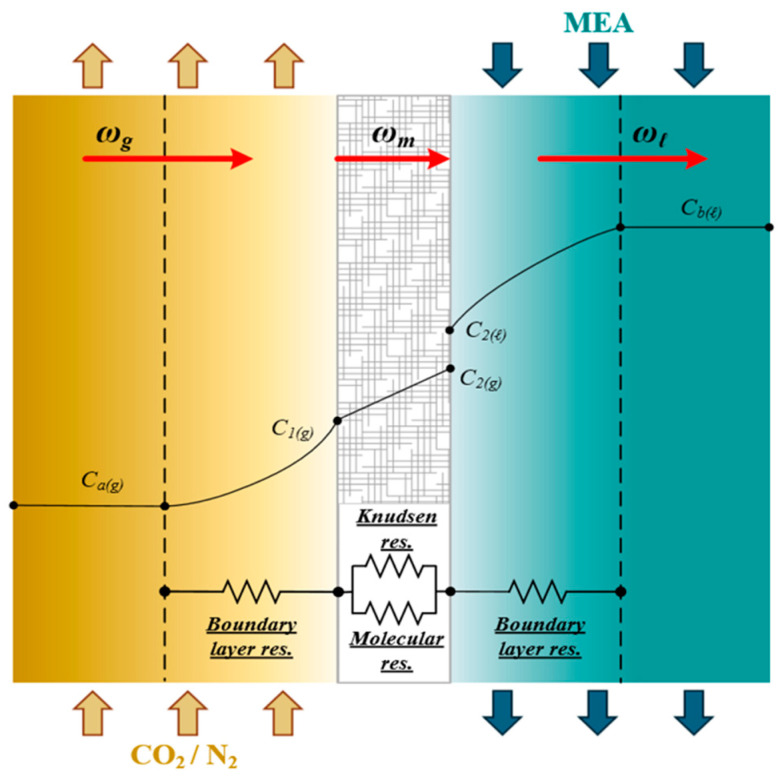
Schematic mass transfer resistances for three mass transfer regions in a gas–liquid membrane contactor. (Brown arrow for N_2_/CO_2_ feed stream, blue arrow for MEA feed stream and red arrow for absorption flux).

The coupled diffusion–reaction pathway in a gas–liquid membrane contactor can be decomposed into three serial transport domains that control CO_2_ transfer from the CO_2_/N_2_ feed to the MEA stream: (a) migration from the bulk gas to the gas-side membrane surface, (b) passage across the membrane pores, and (c) uptake into the liquid phase with simultaneous chemical reaction. These steps are represented with a resistance-in-series formulation using the gas-side coefficient ($k_{a}$), the membrane coefficient ($K_{m}$), and the liquid-side coefficient ($k_{L}=k_{b}/H_{c}$), together with spatial variations in CO_2_ concentration. Figure 4 schematically summarizes the corresponding resistances.

The assumptions adopted in this model are as follows: (1) the membrane gas-absorption system operates under steady-state and isothermal conditions; (2) membrane properties are spatially uniform; (3) the gas phase behaves as an ideal gas; (4) the membrane pores are completely filled with gas; and (5) gas–liquid equilibrium at the interface is governed by Henry’s law. Non-wetting conditions of the membrane were assumed in the mathematical formulation. Here, $C_{a(g)}$ and $C_{b(l)}$ denote the bulk concentrations in the gas feed and MEA liquid solution, respectively, while $C_{1(g)}$ and $C_{2(l)}$ represent the gas- and liquid-phase concentrations at the membrane–liquid interface. Henry’s law, defined by the dimensionless Henry’s constant $H_{c}=C_{\left(g\right)}/C_{\left(l\right)}=1.32$ [21], expresses the equilibrium relationship between the concentration of CO_2_ in the gas phase and its solubility in the liquid phase.

(1) $$P_{2(g)}=C_{2(g)}RT=hC_{2(l)}$$

or

(2) $$H_{c}=\frac{h}{RT}=\frac{c_{2\left(g\right)}}{c_{2\left(l\right)}}$$

An isothermal diffusion–reaction model was then constructed to quantify the CO_2_ absorption rate in the module (Figure 4). In this description, CO_2_ moves sequentially through: (1) gas film diffusion from the bulk gas to the membrane interface; (2) pore transport from the gas-side interface to the liquid-side interface; and (3) liquid film diffusion into the MEA bulk, where reaction consumes dissolved CO_2_. The dusty gas model [17] was adopted to compute absorption flux [25], written in terms of a membrane permeation parameter $\left(c_{m}\right)$ [26] and the transmembrane driving force expressed as a saturation partial-pressure difference (ΔP) [27] between the membrane surfaces on the MEA absorbent and CO_2_/N_2_ gas feed as follows:

(3) $$\omega_{m}=c_{m}\left(P_{1}-P_{2}\right)\frac{1}{M_{w}}={\left.c_{m}\frac{dP}{dC}\;\right|}_{C_{mean}}\left(C_{1\left(g\right)}-C_{2\left(g\right)}\right)\frac{1}{M_{w}}\;\;\;\;\;\;\;\;\;\;\;\;\;\;\;\;=c_{m}RT(C_{1(g)}-{H_{c}K}_{ex}^{\prime }C_{2\left(l\right)})\frac{1}{M_{w}}=K_{m}(C_{1(g)}-{H_{c}K}_{ex}^{\prime }C_{2\left(l\right)})$$

where

(4) $$K_{ex}^{\prime }=K_{ex}[\mathrm{MEA}]/[H^{+}],\;K_{ex}=[{\mathrm{MEACOO}}^{-}]\;[H^{+}]/[CO_{2}][\mathrm{MEA}]=1.25\times 10^{-5}$$

(5) $$c_{m}={\left(\frac{1}{c_{K}}+\frac{1}{c_{M}}\right)}^{-1}={\left\{{\left[1.064\frac{\varepsilon \;r_{p}}{\tau \delta_{m}}{\left(\frac{M_{w}}{RT_{m}}\right)}^{1/2}\right]}^{-1}+{\left[{\left|Y_{m}\right|}_{ln}\frac{D_{m}\varepsilon }{\delta_{m}\tau }\frac{M_{w}}{RT_{m}}\right]}^{-1}\right\}}^{-1}$$

Here, $K_{m}$ denotes the overall membrane mass transfer coefficient; $K_{ex}^{\prime }$ is the reduced equilibrium constant derived from $K_{ex}^{\prime }$ at T=298 K [28]; ${\left|Y_{m}\right|}_{ln}$ is the log mean CO_2_ mole fraction within the membrane; $D_{m}$ the CO_2_ diffusivity in N_2_; $M_{w}$ is the mean molecular weight of the CO_2_/N_2_ mixture; and the tortuosity is taken as τ=1/ε [29].

The CO_2_ absorption flux is primarily governed by the concentration difference across the membrane, which dominates the transmembrane mass flux. This mass flux is controlled by the concentration boundary layers on both bulk streams, membrane properties, and operating conditions. Meanwhile, CO_2_ mass diffusion is driven by the concentration gradient force across the membrane surfaces on both the gas and liquid sides, as shown in Figure 4, and can be expressed as follows:

(6) $$\omega_{g}\;{=k}_{a}\left(C_{a\left(g\right)}-C_{1\left(g\right)}\right)$$

(7) $$\omega_{l}=k_{b}\left(K_{ex}^{\prime }C_{2\left(l\right)}-C_{b\left(l\right)}\right){=k}_{L}\left(K_{ex}^{\prime }H_{c}C_{2(l)}-{H_{c}C}_{b(l)}\right)$$

**Figure 5:**
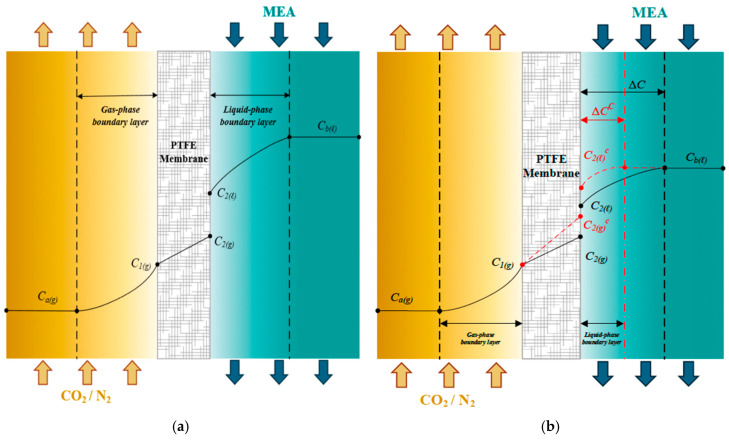
Reduction in mass transfer boundary polarization layers in the absorption contactor module. (**a**) Empty channel; (**b**) Channel with inserting carbon fiber filaments.

Concentration differences between the membrane surfaces and their respective bulk streams generate concentration polarization. This effect can be weakened by using eddy promoters that strengthen the local driving force gradient between surface concentrations, quantified as $\Delta C^{c}=C_{1(g)}^{c}-C_{2(g)}^{c}$ (Figure 5). The concentration polarization coefficient $\gamma_{m}$ is introduced to measure the relative importance of membrane resistance in Figure 5.

The concentration polarization coefficient $\gamma_{m}$ is defined as follows:

(8) $$\gamma_{m}=\frac{C_{1\left(g\right)}-{H_{c}K}_{ex}^{\prime }C_{2(l)}}{C_{a\left(g\right)}-H_{c}C_{b(l)}}$$

Applying mass conservation by matching fluxes across the three intervals ($C_{a\left(g\right)}-C_{1\left(g\right)}),\;(C_{1\left(g\right)}-C_{2\left(g\right)})$, and ($C_{2(l)}-C_{b(l)})$, together with $\omega_{m}=\omega_{g}$ (consistent with Equations (3) and (6)) and $\omega_{m}=\omega_{l}$ (consistent with Equations (3) and (7)), allows solving for the interfacial concentrations ($C_{1(l)}$ and $C_{2(g)}$) and estimating the convective mass transfer coefficients ($k_{a}$ and $k_{b}$) for the gas and MEA streams. Incorporating S-ribbed carbon fiber channels was shown to increase CO_2_ absorption flux by disturbing the near-wall laminar layer and promoting stronger local mixing close to the membrane surface, thereby suppressing concentration polarization.

### 2.2. Mathematical Modeling by Plug Flow Description

**Figure 6:**
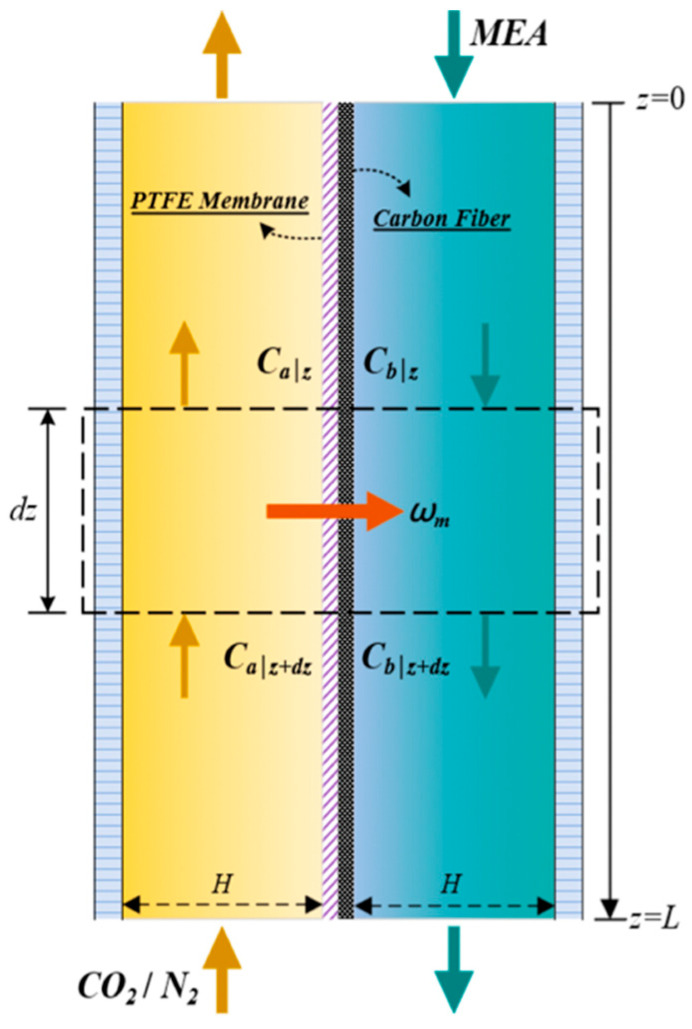
Plug flow description of mass transfer mechanisms within a finite control element (the dashed box).

The one-dimensional governing equations were obtained by formulating an absorption flux representation over a finite control volume and applying steady-state mass conservation along the axial flow direction. Under the plug flow approximation depicted in Figure 6, all axial diffusion terms are neglected, and the description retains only the dominant concentration gradient contribution controlling mass transfer in both the CO_2_/N_2_ and MEA streams as follows:

(9) $$\frac{d\left({Q_{a}C}_{a\left(g\right)}\right)}{\mathrm{dz}}=W\left[k_{m}\gamma_{m}\left(C_{a\left(g\right)}-H_{c}C_{b\left(l\right)}\right)\right]$$

(10) $$\frac{d\left({Q_{b}C}_{b\left(l\right)}\right)}{\mathrm{dz}}=W\left[k_{m}\gamma_{m}\left(C_{a\left(g\right)}-H_{c}C_{b\left(l\right)}\right)-k_{{\mathrm{CO}}_{2}}C_{b\left(l\right)}H\right]$$

The coupled ODE system in Equations (9) and (10) was solved under steady-state conditions for inlet CO_2_ concentrations of 30%, 35%, and 40% by marching along the membrane distillation module shown in Figure S1 of the section of Supplementary Materials. Numerical integration was performed with a Runge–Kutta scheme along the axial coordinate *z* in the flow direction. A fourth order Runge–Kutta method was selected to balance numerical accuracy against computational cost, yielding axial profiles of CO_2_ concentration in both the CO_2_/N_2_ and MEA streams and enabling calculation of the resulting CO_2_ absorption flux and its enhancement. Mass transfer coefficients along the membrane absorption module were obtained from Equation (3) and checked against experiments by iteratively updating $C_{1\left(g\right)}$ and $C_{2(l)}$, starting from an initial guess for $k_{b}$, until the prescribed convergence tolerance was satisfied.

### 2.3. Mass Transfer Enhancement Factor

Comparative tests quantified CO_2_ absorption flux across a range of operating conditions for flat-plate membrane contactor modules operated (i) with S-ribbed carbon fiber turbulence promoters and (ii) with empty channels (no promoters). The resulting improvement in transport performance was characterized using the mass transfer enhancement factor $\alpha^{c}$ [30], which captures the contribution of carbon fiber-filled channels in membrane contactors. Specifically, this factor is defined as the ratio of the mass transfer rate in a promoter-equipped module to the corresponding rate in an empty-channel module, evaluated for different carbon fiber width configurations. The increased mass transfer coefficients were embedded in $\alpha^{c}$ and written through a Sherwood number correlation as follows:

(11) $${\mathrm{Sh}}^{c}=\frac{k_{b}D_{h,promoter}}{D_{b}}=\alpha^{c}{Sh}_{lam}$$

where the correlated Sherwood number ${Sh}^{c}$ is defined the module of inserting various S-ribbed carbon fiber width channels with regressing four dimensionless groups into Buckingham’s π theorem while the Sherwood number ${Sh}_{lam}$ is obtained for the membrane contactor using the non-carbon fiber channel under laminar flow operations with the correlation equation [31] as

(12) $$\alpha^{c}=\frac{{\mathrm{Sh}}^{c}}{{Sh}_{lam}}=f\left(W_{\mathrm{ratio}}\;,\frac{W_{1}}{L},{Re}_{l},\;{Re}_{g},\;{Sc}_{g}\right)=(a+W_{R}){\left(\frac{W_{1}}{L}\right)}^{b}{\left({Re}_{l}\right)}^{c}{\left({Re}_{g}\right)}^{d}{\left({Sc}_{g}\right)}^{e}$$

and

(13) $${Sh}_{lam}={a\left(\frac{W_{1}}{L}\right)}^{b}{\left({Re}_{l}\right)}^{c}{\left({Re}_{g}\right)}^{d}{\left({Sc}_{g}\right)}^{e}$$

where $W_{1}$ is the average carbon fiber width of inserting S-ribbed carbon fiber promoters and $W_{R}$ is the ratio of various carbon fiber promoter widths inserted.

### 2.4. Power Consumption Increment

The present study examines the use of S-ribbed carbon fiber channel geometries as eddy-promoting inserts in membrane contactor systems. While these channels can improve device performance, their benefits are inherently coupled with higher power demand. Consequently, a full appraisal of S-ribbed carbon fiber channels require additional work that explicitly incorporates economic criteria. In particular, it is important to quantify the added frictional losses introduced when S-ribbed carbon fiber channels are embedded within flat-plate membrane contactor modules. In this design, total power consumption includes contributions from both the gas side and the MEA liquid side. These components were assessed using the Fanning friction factor $f_{F}$ for laminar and turbulent regimes [32].

(14) $$H_{i}=Q_{a}\;\rho_{{CO}_{2}}lw_{f,{CO}_{2}}+Q_{b}\;\rho_{MEA}lw_{f,MEA\;},\;i=carbon\;fiber,\;empty$$

(15) $${lw}_{f,\mathrm{MEA}}=\frac{2f_{F,MEA}{\bar{v}}_{MEA}^{2}L}{D_{h,MEA}}$$

(16) $${lw}_{f,{CO}_{2}}=\frac{2f_{F,{CO}_{2}}{\bar{v}}_{{CO}_{2}}^{2}L}{D_{h,{CO}_{2}}}$$

**Figure 7:**
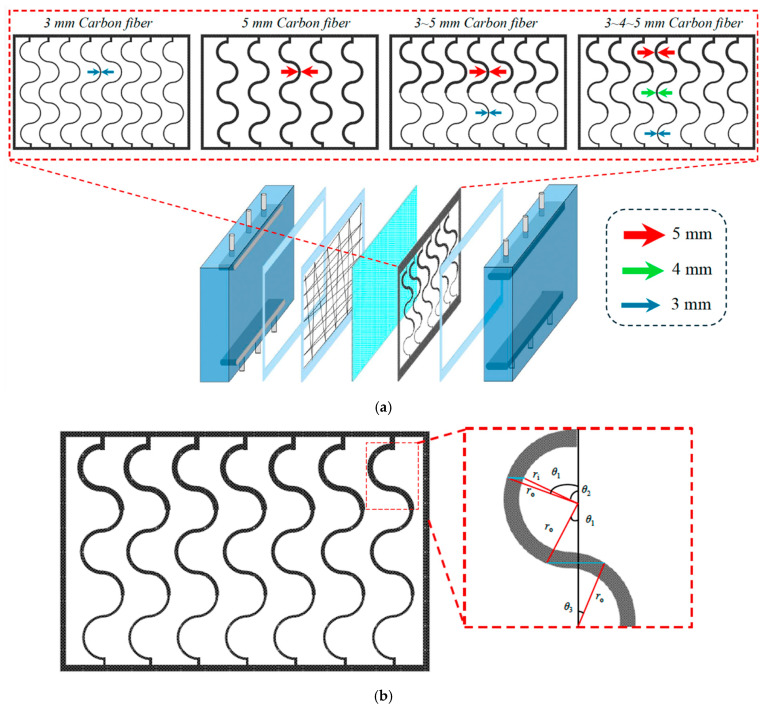
The average carbon fiber width of S-ribs carbon fiber turbulence promoters: (**a**) 3 mm and 5 mm uniform promoter widths, 3–5 mm and 3–4–5 mm ascending or descending promoter widths; (**b**) demonstration of calculated average carbon fiber width.

The total power consumption comprises pressure drops arising from frictional losses in both feed streams, as described by Equation (14), for a flat-plate membrane contactor of known channel length. The equivalent hydraulic diameters, $D_{h,MEA}$ and $D_{h,CO_{2}}$, for modules embedded with S-ribbed carbon fiber turbulence promoters and for modules with empty channels on the MEA and CO_2_/N_2_ feed sides, respectively, were calculated under various hydraulic width configurations. These configurations include uniform promoter widths, ascending promoter widths, and descending promoter widths as illustrated in Figure 7.

The average velocity and equivalent hydraulic diameter of each flow channel are calculated as follows:

(17) $${\bar{v}}_{CO_{2}}=\frac{Q_{a}}{Wd}\;,\;{\bar{v}}_{MEA}=\frac{Q_{b}}{Wd-D_{1}W_{1}N_{1}}$$

(18) $${De}_{h,CO_{2}}=\frac{4\left(\mathrm{Wd}\right)}{2(W+d)},\;{\;\;De}_{h,MEA}=\frac{4\left(Wd-D_{1}W_{1}N_{1}\right)}{2\left(W+d+D_{1}N_{1}\right)}$$

The hydraulic equivalent diameters ${De}_{h,MEA}$ and ${De}_{h,CO_{2}}$ for the modules—with embedded S-ribbed carbon fiber turbulence promoters on the MEA feed stream and CO_2_/N_2_ gas feed stream, respectively. The Fanning friction factor can be estimated using a correlation based on the aspect ratio of the channel (α=d/W) [33]:

(19) $$f_{F,h}=\frac{C}{{Re}_{h}},f_{F,c}=\frac{C}{{Re}_{c}}$$

(20) $$C=24\left(1-1.3553\alpha +1.9467\alpha^{2}-1.7012\alpha^{3}+0.9564\alpha^{4}-0.2537\alpha^{5}\right)$$

The percentage increment in power consumption for the module with inserting S-ribbed carbon fiber channel as compared to the module of using non-S-ribbed carbon fiber channel is illustrated as the relative extents $I_{P}$:

(21) $$I_{P}=\frac{H_{carbon}-H_{empty}}{H_{empty}}\times 100\%$$

where the subscripts of *carbon* and *empty* represent the modules with/without S-ribbed carbon fiber channels, respectively.

## 3. Membrane Absorption Experiments

**Figure 8:**
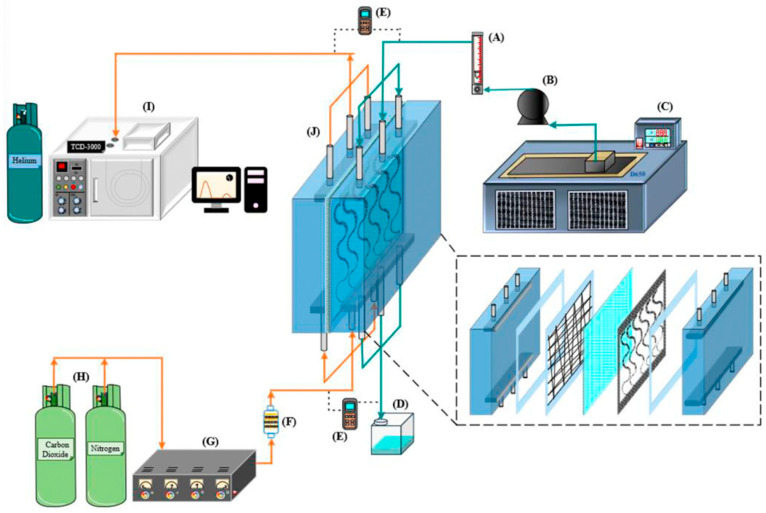
Schematic diagram of fabrication structure and experimental setup for the membrane absorption system of the S-ribs carbon fiber-filled channel: (A) flowmeter; (B) pump; (C) thermostatic tank; (D) waste bucket; (E) temperature sensor; (F) gas mixing adapter; (G) gas mass flow controller; (H) gas cylinder; (I) chromatograph; (J) membrane absorption module.

The schematic configuration and fabrication details of the flat-plate gas–liquid membrane contactor used for CO_2_ absorption with an MEA absorbent (Uni-Onward Corp., New Taipei, Taiwan) are illustrated in Figure 8.

**Figure 9:**
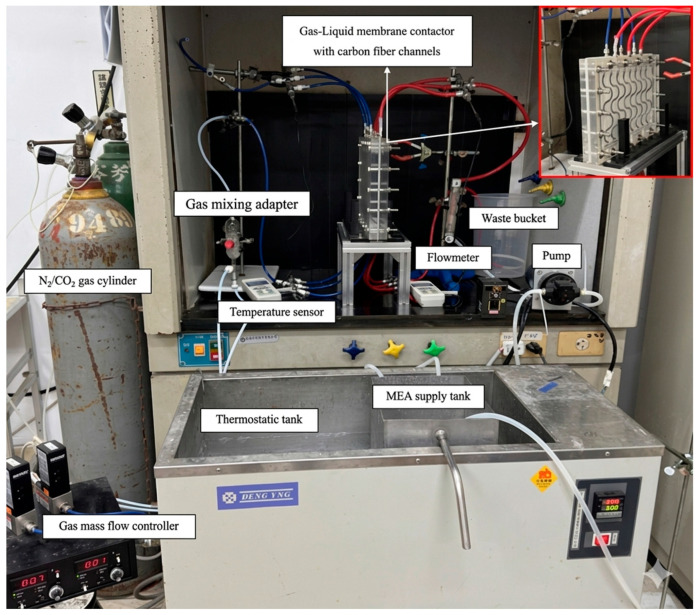
Photographic images of the experimental apparatus for the flat-plate gas/liquid membrane contactor.

The membrane absorption module consists of an acrylic plate with a ditched structure, a spacer layer, and a membrane, and it was thermally insulated with styrofoam prior to final assembly using screws and nuts. Channel-forming spacers were fabricated from silicon dioxide (silicon gel). Two configurations were prepared: one module incorporated an S-ribbed carbon fiber-filled structure in the MEA flow channel, while the comparison module used an empty channel; in the latter, a 0.1 mm nylon fiber was wound as a support wrap around the membrane surface to provide reinforcement and prevent bending or wrinkling under the CO_2_/N_2_ feed stream. A photograph of the experimental apparatus for the flat-plate gas membrane absorption system is shown in Figure 9, in which acrylic plates served as the outer walls of the parallel-plate channel. Experimental runs were conducted by introducing a CO_2_/N_2_ gas mixture at 293 K into the flat-plate membrane absorption contactor, while an aqueous MEA absorbent solution flowed through the S-ribbed carbon fiber-filled channel. The diluted 30 wt.% MEA solution was circulated using a pump (51K40RA-A, ASTK, New Taipei, Taiwan) from a thermostat (G-50, DENG YNG, New Taipei, Taiwan), and the solution temperature was monitored at 303 K using a thermometer (TM-946, Lutron, New Taipei, Taiwan). The MEA flow rate was regulated by a flowmeter (MB15GH-4-1, Fong-Jei, New Taipei, Taiwan) over a range of 5–10 cm^3^/s (5.0, 6.67, 8.33, and 10.0 cm^3^/s). After reacting with the CO_2_/N_2_ gas mixture, the CO_2_-loaded MEA solution was discharged into a waste container under one-through operation. Gas mixtures containing 30%, 35%, and 40% CO_2_ (balance N_2_) of industrial-grade purity were prepared in a gas mixing tank (400-0701-FLS, Cole-Parmer Company, Vernon Hills, IL, USA) to ensure complete mixing. The composition of the well-mixed gas feed was adjusted and supplied to the empty gas channel of the membrane module until steady-state conditions were reached using a mass flow controller (5850E Series, Brooks Instrument, Hatfield, PA, USA) at a flow rate of 5 cm^3^/s. The gas mixture subsequently diffused through the microporous hydrophobic membrane pores of the membrane absorption contactor. At steady state, the outlet gas from the flat-plate membrane module was collected and directed to a column-heating system for rapid heating of the sample collection capillary tube. The CO_2_ concentration was then analyzed using a gas chromatograph (Model HY 3000, China Chromatograph Co., Ltd., New Taipei, Taiwan) equipped with a thermal conductivity detector (TCD) and helium as the carrier gas, with data recorded on a personal computer. Measurement reproducibility was generally maintained within a deviation of 5%, which was used to evaluate CO_2_ absorption efficiency.

**Figure 10:**
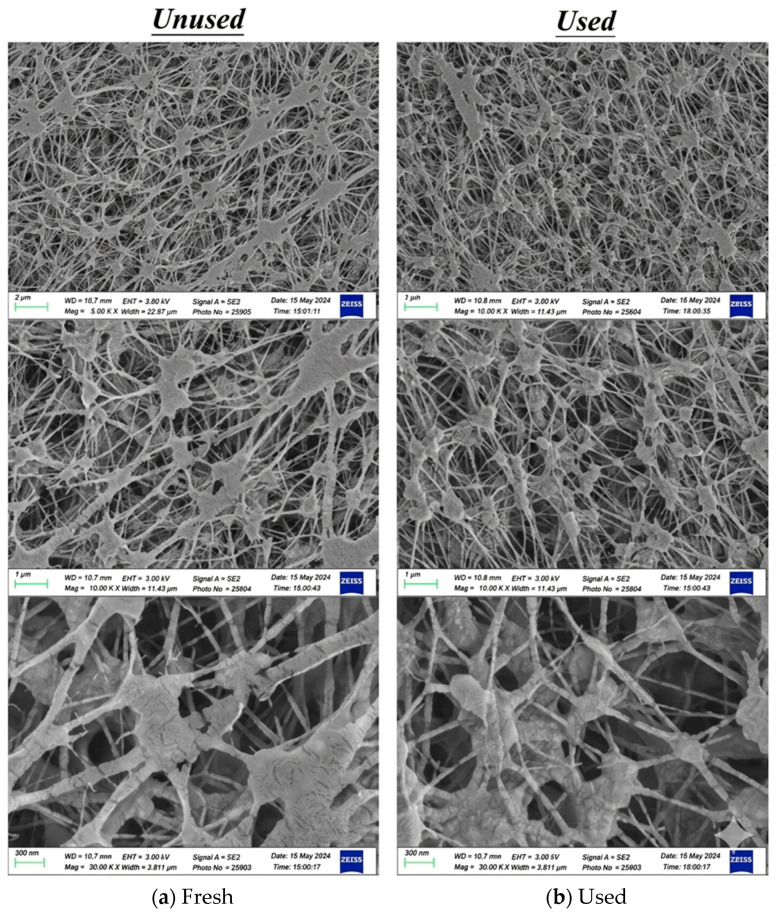
SEM images of the PTFE/PP membrane for fresh and used membranes of experimental runs. (**a**) Fresh; (**b**) Used.

**Figure 11:**
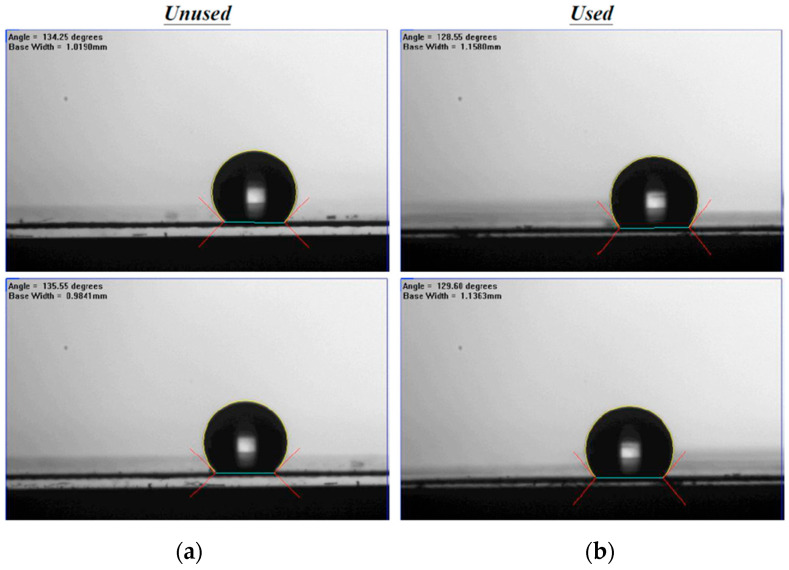
Sessile drop contact angles of PTFE/PP membranes. (**a**) Unused; (**b**) Used.

The S-ribbed carbon fiber turbulence promoters (TORAY T300B, San Li Composites, Co. Ltd., Taichung, Taiwan) were selected for their rigidity, chemical resistance, and low thermal resistance, serving both as mechanical supports to reduce membrane vibration and as mixing enhancers to improve mass transfer. The promoters have a tensile strength of 3530 MPa and a thermal conductivity of 0.105 J/cm s °C. Because the S-rib geometry partially obstructed the effective permeation area (about 13%), this reduction was explicitly included in performance calculations. The promoters were manufactured and immersed in MEA solution for a 48 h durability assessment, confirming stability and resistance without observable degradation during experiments. In addition, PTFE/PP composite membranes were characterized before and after operation in terms of morphology and water contact angles. Figure 10 presents SEM images (Zeiss Sigma 300, Jena, Germany) of fresh versus used membranes, and hydrophobicity was evaluated the contact angle using FTA-125 (First Ten Angstrom, Inc., Newark, CA, USA). As indicated in Figure 11, measured contact angles were 128–135° (122 ± 3.0°), consistent with a hydrophobic membrane surface.

## 4. Results and Discussions

### 4.1. CO_2_ Absorption Flux Improvement by Embedding Various S-Ribbed Carbon Fiber Widths

**Figure 12:**
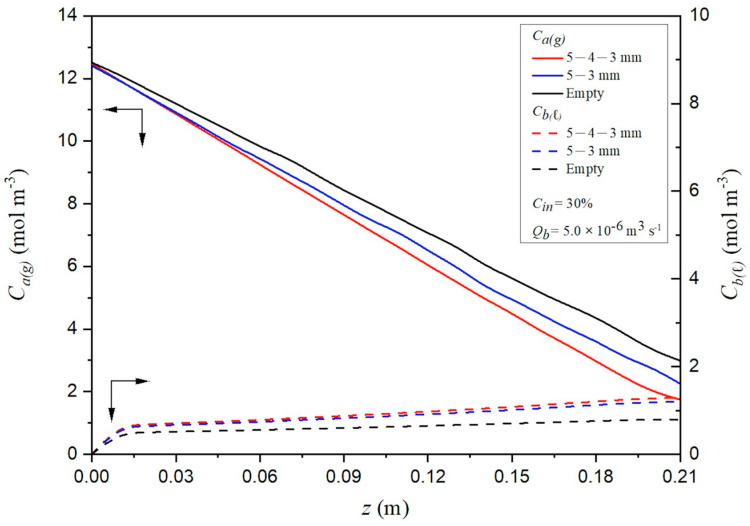
Effects of descending carbon fiber width and empty channels on concentration distributions along the module ($C_{in}=30\%$).

Bulk concentration profiles for both the CO_2_/N_2_ gas stream and the MEA liquid stream were obtained by numerically solving the one-dimensional model. Figure 12 provides an illustrative case using a 30% inlet CO_2_ feed concentration and a 0.3 L/min MEA flow rate, reporting axial distributions for (i) a channel with descending carbon fiber width and (ii) an empty channel. For the descending configuration, the model predicts that bulk concentrations progressively decrease along the flow direction, which corresponds to a weakening of the driving force temperature gradients. The results further show that the concentration differences across the two membrane surfaces are larger when S-ribbed carbon fiber turbulence promoters are present than when the channel is empty. The amplified surface-to-surface concentration gradients promote greater vapor transport through the membrane, thereby increasing the absorption flux ultimately taken up by the MEA solution.

Application of Runge–Kutta numerical scheme in a marching solution procedure to Equations (9) and (10) was conducted to estimate the CO_2_ concentrations distributions in the CO_2_/N_2_ and MEA absorbent feed streams as well as CO_2_ absorption flux for implementing various S-ribbed carbon fiber widths. The range and limits of deviation between theoretical predictions and experimental results for the experimental measurements are calculated by using the following definition of accuracy deviation [34]:

(22) $$E\;(\%)=\frac{1}{N_{exp}}\sum_{j=1}^{N_{exp}}\frac{\left|\omega_{theo,j}-\omega_{\exp,j}\right|}{\omega_{\exp,j}}$$

where $N_{exp}$, $\omega_{theo,j}$, and $\omega_{\exp,j}$ are the number of experimental runs, theoretical predictions, and experimental results of absorption fluxes, respectively. The accuracy deviations in both ascending and descending S-ribbed carbon fiber width operations are shown in Table 1 and Table 2, respectively. The agreement of experimental results that deviate from theoretical predictions is fairly good within $1.1\times 10^{-3}\leq E\leq 3.48\times 10^{-2}$.

Embedding S-ribbed carbon fiber-filled channels resulted in a marked improvement in CO_2_ absorption flux compared with non-S-ribbed carbon fiber-filled modules under identical operating conditions. Operation with various S-ribbed carbon fiber width configurations contributes to enhanced turbulence intensity and elevated shear near the membrane surfaces, leading to an almost twofold increase in CO_2_ absorption flux. This enhancement is attributable to the effective reduction in mass transfer resistances across the concentration boundary layers in the MEA feed streams, as confirmed by the experimental observations [35]. Two curved geometries of S-ribbed carbon fiber turbulence promoters were used in gas/liquid membrane contactors, with ascending (3–5 mm and 3–4–5 mm) and descending (5–3 mm and 5–4–3 mm) configurations at various average equivalent hydraulic widths. All promoters occupied approximately 13% of the hydrophobic membrane surface area, allowing for a comparison of CO_2_ absorption fluxes in this study. The incorporation of turbulence promoters aimed to enhance turbulence intensity, effectively reducing the concentration polarization effect near the membrane surfaces. This was achieved by increasing velocities and vortices, which enhanced shear stress on the membrane surface, ultimately improving CO_2_ absorption efficiency and device performance. Notably, the insertion of 5–4–3 mm S-ribbed carbon fiber turbulence promoters, with their more curvature changes, led to significantly higher CO_2_ absorption flux compared to the 5–4–3 mm promoters. This suggests that operating a channel filled with descending 5–4–3 mm turbulence promoters generated higher turbulence intensity, which reduced concentration polarization resistance and further enhanced CO_2_ absorption flux. The CO_2_ absorption fluxes for modules incorporating ascending and descending S-ribbed carbon fiber width channels are presented in Figures S2 and S3 of the section of Supplementary Materials, respectively, including both experimental results and theoretical predictions. Notably, the descending S-ribbed carbon fiber width channel consistently achieved superior absorption flux performance compared with the ascending configuration, as demonstrated in Figures S2 and S3 of the section of Supplementary Materials. As expected, increases in both MEA feed flow rate and inlet CO_2_ concentration resulted in higher absorption fluxes.

The absorption flux improvements $I_{E}^{as}$ and $I_{E}^{des}$ were illustrated by calculating the percentage increase in the device by inserting both ascending and descending S-ribbed carbon fiber widths based on the device with an empty channel as follows:

(23) $$I_{E}^{as}(\%)=\frac{\omega_{carbon}^{as}-\omega_{empty}}{\omega_{empty}}\times 100,\;\mathrm{module}\;\mathrm{with}\;\mathrm{ascending}\;\mathrm{widths}$$

(24) $$I_{E}^{des}(\%)=\frac{\omega_{carbon}^{des}-\omega_{empty}}{\omega_{empty}}\times 100,\;\mathrm{module}\;\mathrm{with}\;\mathrm{descending}\;\mathrm{widths}$$

where the subscripts *carbon* and *empty* represent the channels with/without S-ribbed carbon fiber widths, respectively, while the superscripts *as* and *des* represent ascending and descending S-ribbed carbon fiber-filled configuration, respectively. The theoretical predictions of the CO_2_ absorption flux improvements $I_{E}^{as}$ and $I_{E}^{des}$ for various MEA feed flow rates and inlet feed CO_2_ concentrations under ascending and descending S-ribbed carbon fiber-filled channels were calculated and presented in Table 3 and Table 4, respectively, compared with the absorption fluxes of the modules with non-S-ribbed carbon fiber-filled channels.

Furthermore, as summarized in Table 3 and Table 4, a maximum absorption flux improvement of up to 44.91% was achieved relative to modules equipped with non-S-ribbed carbon fiber-filled channels. Overall, the incorporation of S-ribbed carbon fibers within the flow channel exhibits strong potential for absorption flux enhancement, with descending S-ribbed carbon fiber width channels providing more pronounced performance improvements than ascending configurations.

### 4.2. Further CO_2_ Absorption Flux Enhancement

**Figure 13:**
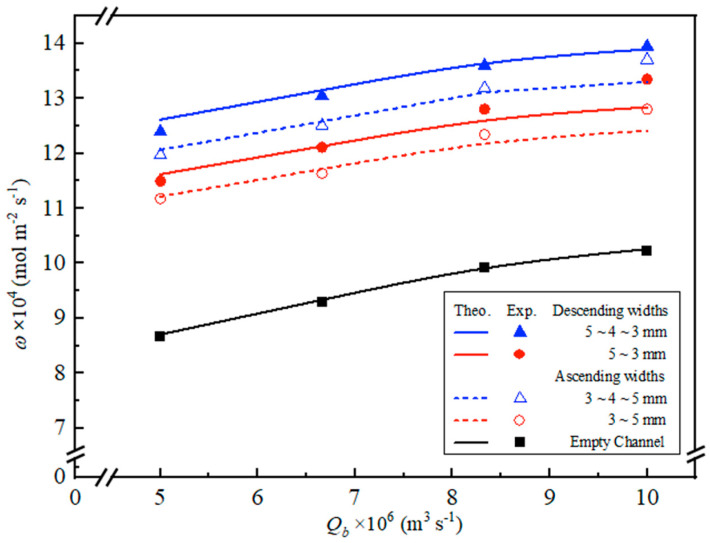
Comparisons of theoretical CO_2_ absorption flux with/without carbon fiber-filled channels ($C_{in}=40\%$).

**Figure 14:**
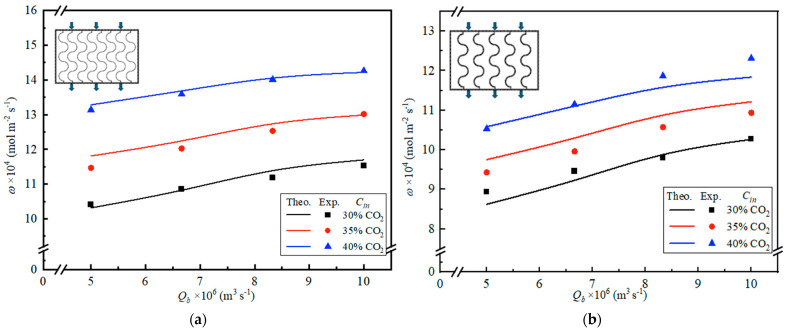
Effects of MEA flow rate and inlet CO_2_ feed concentration on CO_2_ absorption flux uniform carbon fiber widths. (**a**) 3 mm carbon fiber width; (**b**) 5 mm carbon fiber width.

Figure 13 and Figure 14 summarize the additional gains in CO_2_ absorption flux obtained by employing S-ribbed carbon fiber channels with different width patterns, including ascending/descending arrangements and uniform width designs, respectively. The results indicate that a varying-width S-ribbed carbon fiber layout is technically more advantageous, delivering noticeably greater absorption flux than either constant-width S-ribbed carbon fiber channels or an empty-channel baseline.

The further absorption flux enhancement $E_{promoter}$ of CO_2_ capture by implementing S-ribbed carbon fiber width-filled membrane contactors under various descending carbon fiber widths is calculated based on the device of the same working dimensions performed with 5 mm uniform carbon fiber width as follows:

(25) $$\begin{array}{ll} E_{promoter}\left(\%\right) & =\frac{\omega_{carbon}^{des}-\omega_{carbon}^{con}}{\omega_{carbon}^{con}}\times 100=\left[\frac{\left(\omega_{carbon}^{des}-\omega_{empty})-(\omega_{carbon}^{con}-\omega_{empty}\right)}{\omega_{empty}}\right]\left(\frac{\omega_{empty}}{\omega_{carbon}^{con}}\right)\times 100\\ & =\left(I_{E}^{des}-I_{E}^{con}\right)/\left(1+I_{E}^{con}\right)\times 100 \end{array}$$

where $J_{carbon}^{con}$ and $J_{carbon}^{des}$ represent the CO_2_ absorption fluxes measured in the present study for modules fitted with a constant (uniform) carbon fiber width and with descending carbon fiber width arrangements, respectively. To quantify the incremental benefit of adopting descending widths, the percentage increase in absorption flux enhancement was calculated relative to both the uniform 5 mm carbon fiber width channel and the empty-channel module; the resulting comparisons are reported in Table 5.

This increase in absorption flux enhancement is achieved through the combined application of two scaling strategies, the insertion of carbon fibers and the use of descending carbon fiber width configurations, which jointly increase turbulence intensity and, consequently, enhance the convective mass transfer coefficient. The additional gain (in percentage) in absorption flux enhancement achieved by using descending widths was computed and summarized in Table 5 for comparison, which is comparable to both a uniform 5 mm carbon fiber width channel and an empty-channel baseline. In particular, the largest incremental improvement was 43.04%, observed for the descending 5–4–3 mm S-ribbed carbon fiber width channel when operating at 40% inlet CO_2_ and an MEA feed flow rate of $10.0\;\times \;10^{-6}$ m^3^/s MEA feed flow rate. The magnitude of this further enhancement increases as inlet CO_2_ concentration rises, but it diminishes as the MEA feed flow rate increases.

### 4.3. Correlated Sherwood Numbers

The mass transfer coefficients used to estimate CO_2_ absorption flux for S-ribbed carbon fiber-filled modules operated in both ascending and descending modes were cast in Sherwood number form, consistent with the definition in Equation (11). They were obtained from the theoretical framework by treating the empty-channel module as the baseline (reference) configuration. For the empty-channel module, the Sherwood number shows a linear correspondence with experimental measurements, as described by Equation (13) and corroborated in Figures S4 and S5 of the section of Supplementary Materials. As anticipated, insertion of the S-ribbed carbon fiber structures produced larger convective mass transfer coefficients, which is manifested as Sherwood numbers exceeding those of the empty-channel case and summarized in Equations (26) and (27) and illustrated in Figure S4 for a 35% inlet CO_2_ feed concentration.

The mass transfer enhancement factor, αᶜ, defined in Equation (12), was correlated via regression analysis by formulating the normal equations for least-squares parameter estimation. The resulting squared correlation coefficients ($R^{2}$) ranged from 0.94 to 0.97 for the S-ribbed carbon fiber-filled modules, with deviations between correlated and experimental Sherwood numbers remaining within 10%, as illustrated in Figure S5.

(26) Shlam=4.88W1L−0.82Rel0.42Reg−3.80Scg1.93

(27) Shc=(8.22+Wr)W1L−0.74Rel0.32Reg−1.99Scg0.55

**Figure 15:**
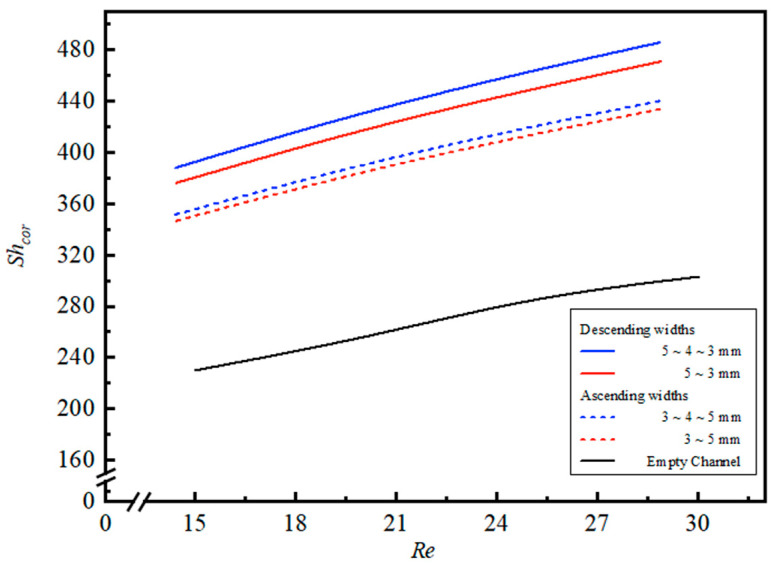
Influences of both descending and ascending carbon fiber widths on corrected Sherwood numbers ($C_{in}=35\%$).

In addition, the Sherwood number correlations presented in Figure 15 for a 35% inlet CO_2_ feed concentration show that modules equipped with descending S-ribbed carbon fiber width channels achieve larger correlated Sherwood numbers than both ascending width designs and the empty-channel case. This increase is attributed to more effective disruption of the near-wall concentration boundary layer, which strengthens convective mass transfer and consequently elevates CO_2_ absorption flux.

### 4.4. Power Consumption Increment by Inserting S-Ribbed Carbon Fiber Turbulence Promoters

**Figure 16:**
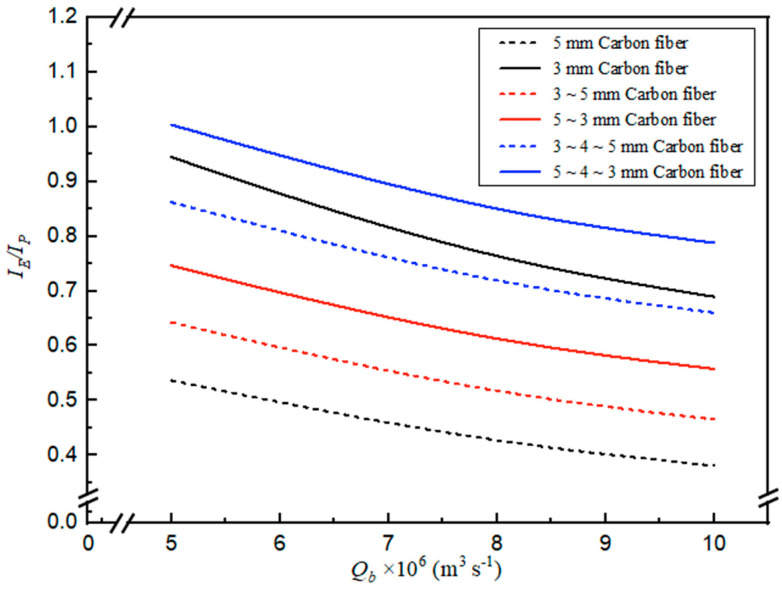
Effects of MEA flow rate and carbon fiber widths on $I_{E}/I_{P}$ ($C_{in}=35\%$).

Identifying operating conditions that balance absorption flux gains against added energy demand is essential for optimizing carbon fiber-filled channel insertion. From an economic perspective, this trade-off was evaluated using the indicator $I_{E}/I_{P}$. Figure 16 presents $I_{E}/I_{P}$ results for a 40% inlet CO_2_ feed concentration, with MEA feed flow rate and carbon fiber width configuration treated as key parameters. The analysis indicates that selecting an appropriate S-ribbed carbon fiber width arrangement of the most notable descending width designs produces larger $I_{E}/I_{P}$ values, implying absorption flux benefit (per unit power) increases, overwhelmingly improving technical feasibility at a given power consumption level (Figure 16). The comparison reveals that, although a higher Sherwood number was obtained for the constant 3 mm carbon fiber width module than for the descending carbon fiber width modules (Figure S4), the corresponding $I_{E}/I_{P}$ ratios for the various carbon fiber width configurations exhibit an inverse ranking in Figure 16 when evaluated from an economic perspective. In other words, the relative percentage increase in absorption flux enhancement exceeds that of the associated energy consumption increment, indicating that appropriately varying carbon fiber widths can achieve higher absorption flux while mitigating undesirable increases in frictional losses. The results further demonstrate that descending carbon fiber width configurations produce larger $I_{E}/I_{P}$ values than those obtained with constant or ascending carbon fiber width modules, implying the establishment of stronger concentration driving force gradients within the descending configurations. However, it was also observed that, at higher MEA flow rates, the increase in CO_2_ absorption flux could not offset the corresponding increase in power consumption.

## 5. Conclusions

The model-based CO_2_ absorption flux predictions were computed and then benchmarked against experimental measurements collected across a range of MEA flow rates, inlet CO_2_ feed concentrations, and S-ribbed carbon fiber hydraulic width patterns operated in both ascending and descending modes. Based on these cross-condition comparisons of flux enhancement produced by inserting S-ribbed carbon fiber turbulence promoters into the MEA feed channels, the following conclusions are drawn:

1. Varying S-ribbed carbon fiber widths resulted in greater absorption flux enhancement compared with modules employing uniform S-ribbed carbon fiber-filled channels and non-S-ribbed (empty) channels. This improvement is primarily attributed to an increase in the overall mass transfer coefficient within the MEA absorbent feed stream.
2. The insertion of 3 mm S-ribbed carbon fiber promoters into the MEA absorbent feed channel produced substantial increases in absorption flux, achieving a maximum improvement of 44.91% under the descending 5–4–3 mm configuration relative to the empty-channel module (without S-ribbed carbon fiber promoters).
3. Among the investigated flow characteristics associated with various S-ribbed carbon fiber widths, descending width configurations exhibited a pronounced positive influence on absorption flux, owing to enhanced concentration gradients induced by properly adjusted width variations.
4. Modules utilizing descending S-ribbed carbon fiber width configurations consistently demonstrated higher absorption flux enhancements than those employing uniform width configurations. Moreover, the descending 5–4–3 mm S-ribbed carbon fiber width channel achieved a favorable $I_{E}/I_{P}$ ratio while maintaining relatively lower specific power consumption.

Two array configurations of turbulence promoters were investigated for gas/liquid membrane contactors and compared with a uniform S-ribbed carbon fiber width channel and an empty channel in the present study. This work specifically focuses on intensifying turbulence within a gas/liquid PTFE/PP membrane contactor for CO_2_ absorption through the incorporation of varying S-ribbed carbon fiber width channels. The correlated Sherwood number expression used to evaluate the convective mass transfer coefficients, derived from the theoretical model, demonstrates strong potential for significantly mitigating concentration polarization and enhancing absorption flux. Further investigations are recommended to explore alternative geometric shapes and array configurations of S-ribbed carbon fiber promoters or other eddy promoters, particularly when economic feasibility is taken into consideration.

## Figures and Tables

**Table 1:** The accuracy deviation between theoretical predictions and experimental results (Ascending).

$C_{in}$ (%)	$Q_{b}\;\times \;10^{6}$ (m^3^ s^−1^)	Ascending Carbon Fiber Width Operations (mol m^−2^ s^−1^)					
		3–5 mm			3–4–5 mm		
		$\omega_{exp}\;\times \;10^{4}$	$\omega_{theo}\;\times \;10^{4}$	E (%)	$\omega_{exp}\;\times \;10^{4}$	$\omega_{theo}\;\times \;10^{4}$	E (%)
30	5.0	9.29	9.03	2.83	9.86	9.53	3.48
	6.67	9.69	9.55	1.48	10.18	10.08	1.00
	8.33	10.10	10.23	1.21	10.65	10.80	1.34
	10.0	10.50	10.50	0.05	11.03	11.09	0.51
35	5.0	10.09	10.22	1.23	10.70	10.81	1.06
	6.67	10.50	10.70	1.93	11.15	11.32	1.47
	8.33	11.07	11.35	2.43	11.65	11.98	2.80
	10.0	11.54	11.59	0.41	12.34	12.24	0.82
40	5.0	11.18	11.21	0.34	11.97	12.06	0.78
	6.67	11.63	11.71	0.67	12.51	12.58	0.60
	8.33	12.34	12.22	0.98	13.19	13.11	0.62
	10.0	12.80	12.41	3.15	13.70	13.30	3.00

**Table 2:** The accuracy deviation between theoretical predictions and experimental results (Descending).

$C_{in}$ (%)	$Q_{b}\;\times \;10^{6}$ (m^3^ s^−1^)	Descending Carbon Fiber Width Operations (mol m^−2^ s^−1^)					
		5–3 mm			5–4–3 mm		
		$\omega_{exp}\;\times \;10^{4}$	$\omega_{theo}\;\times \;10^{4}$	E (%)	$\omega_{exp}\;\times \;10^{4}$	$\omega_{theo}\;\times \;10^{4}$	E (%)
30	5.0	9.57	9.26	3.38	10.11	9.90	2.18
	6.67	9.94	9.79	1.53	10.47	10.46	0.11
	8.33	10.36	10.47	1.08	10.87	11.17	2.64
	10.0	10.73	10.75	0.20	11.26	11.46	1.73
35	5.0	10.47	10.60	1.23	11.16	11.09	0.66
	6.67	10.86	11.09	2.07	11.60	11.60	0.02
	8.33	11.37	11.77	3.40	12.02	11.92	0.83
	10.0	11.91	12.03	0.93	12.62	12.29	2.69
40	5.0	11.49	11.62	1.07	12.39	12.61	1.75
	6.67	12.11	12.13	0.16	13.04	13.15	0.80
	8.33	12.80	12.65	1.24	13.60	13.69	0.66
	10.0	13.35	12.84	3.96	13.94	13.89	0.37

**Table 3:** Effects of ascending carbon fiber width operations on absorption flux improvements.

$C_{in}$ (%)	$Q_{b}\;\times \;10^{6}$ (m^3^ s^−1^)	Ascending Carbon Fiber Width Operations (mol m^−2^ s^−1^)				
		Empty Channel	3–5 mm		3–4–5 mm	
		$\omega_{empty}\times 10^{4}$	$\omega_{carbon}^{as}\times 10^{4}$	$I_{E}^{as}$ (%)	$\omega_{carbon}^{as}\times 10^{4}$	$I_{E}^{as}$ (%)
30	5.0	7.77	9.03	16.24	9.53	22.65
	6.67	8.46	9.55	12.96	10.08	19.23
	8.33	9.31	10.23	9.80	10.80	15.94
	10.0	9.71	10.50	8.11	11.09	14.16
35	5.0	8.22	10.22	24.34	10.81	31.55
	6.67	8.84	10.70	21.05	11.32	27.99
	8.33	9.62	11.35	17.96	11.98	24.58
	10.0	9.97	11.59	16.22	12.24	22.71
40	5.0	8.70	11.21	28.85	12.06	38.59
	6.67	9.34	11.71	25.47	12.58	34.77
	8.33	9.96	12.22	22.69	13.11	31.57
	10.0	10.26	12.41	21.00	13.30	29.70

**Table 4:** Effects of descending carbon fiber width operations on absorption flux improvements.

$C_{in}$ (%)	$Q_{b}\;\times \;10^{6}$ (m^3^ s^−1^)	Descending Carbon Fiber Width Operations (mol m^−2^ s^−1^)				
		Empty Channel	5–3 mm		5–4–3 mm	
		$\omega_{empty}\times 10^{4}$	$\omega_{carbon}^{des}\times 10^{4}$	$I_{E}^{des}$ (%)	$\omega_{carbon}^{des}\times 10^{4}$	$I_{E}^{des}$ (%)
30	5.0	7.77	9.26	19.16	9.90	27.39
	6.67	8.46	9.79	15.75	10.46	23.65
	8.33	9.31	10.47	12.43	11.17	19.94
	10.0	9.71	10.75	10.67	11.46	18.00
35	5.0	8.22	10.60	28.91	11.09	34.93
	6.67	8.84	11.09	25.43	11.60	31.17
	8.33	9.62	11.77	22.42	11.92	25.25
	10.0	9.97	12.03	20.58	12.29	23.21
40	5.0	8.70	11.62	33.50	12.61	44.91
	6.67	9.34	12.13	29.91	13.15	40.84
	8.33	9.96	12.65	26.94	13.69	37.40
	10.0	10.26	12.84	25.17	13.89	35.45

**Table 5:** Theoretical predictions of further absorption flux enhancement in the module with descending S-ribbed carbon fiber width channels.

$C_{in}$ (%)	$Q_{b}\;\times \;10^{6}$ (m^3^ s^−1^)	Descending Carbon Fiber Widths				
		Uniform 5 mm Width	5–3 mm		5–4–3 mm	
		$I_{E}^{con}$ (%)	$E_{promoter}$ (%)	$I_{E}^{des}$ (%)	$E_{promoter}$ (%)	$I_{E}^{des}$ (%)
30	5.0	24.77	7.05	22.48	13.12	29.42
	6.67	21.17	5.02	17.18	10.63	23.44
	8.33	17.93	5.66	11.68	10.94	17.26
	10.0	16.03	4.43	11.47	9.65	17.04
35	5.0	26.92	10.94	28.41	18.35	36.99
	6.67	25.98	9.01	23.42	16.39	31.77
	8.33	23.41	7.56	18.59	13.62	25.28
	10.0	21.41	8.93	20.14	15.36	27.24
40	5.0	30.41	9.13	32.68	14.39	43.04
	6.67	27.36	8.57	30.27	13.04	40.32
	8.33	25.17	7.87	29.18	13.60	37.20
	10.0	24.25	8.38	30.67	13.94	36.51

## Data Availability

Data is contained within the article.

## Supplementary Materials

The following supporting information can be downloaded at https://www.mdpi.com/article/10.3390/membranes16050162/s1, Figure S1: Flow chart for determining concentrations in both CO_2_/N_2_ and MEA feed streams; Figure S2: Effects of MEA flow rate and inlet CO_2_ feed concentration on CO_2_ absorption flux under ascending and descending carbon-fiber-width configurations; Figure S3: Effects of MEA flow rate and inlet CO_2_ feed concentration on CO_2_ absorption flux under ascending and descending carbon-fiber-width configurations; Figure S4: Comparison of correlated and experimental Sherwood numbers for empty channel and S-ribbed carbon-fiber channel under both descending and ascending operations (*C*_*in*_ = 35%); Figure S5: Comparisons between the correlated and experimental Sherwood numbers.

## Abbreviations

C	Concentration (mol m^−3^)
$C_{mean}$	Mean value of C (mol m^−3^)
$c_{K}$	Membrane coefficient based on the Knudsen diffusion model (mol m^−2^ pa^−1^ s^−1^)
$c_{M}$	Membrane coefficient based on the molecular diffusion model (kg m^−2^ Pa^−1^ s^−1^)
$c_{m}$	Membrane permeation coefficient (mol m^−2^ pa^−1^ s^−1^)
$D_{a}$	Diffusion coefficient of CO_2_ in gas feed stream (m^2^ s^−1^)
$D_{b}$	Diffusion coefficient of CO_2_ in MEA absorbent feed stream (m^2^ s^−1^)
$D_{m}$	Diffusion coefficient of CO_2_ in N_2_ (m^2^ s^−1^)
$D_{1}$	Carbon fiber thickness (m)
d	Channel height (m)
$d_{h}$	Equivalent hydraulic diameter of channel (m)
E	Deviation of experimental results from the theoretical predictions
$E_{promoter}$	Further absorption flux enhancement
$f_{F}$	Fanning friction factor
$H_{c}$	Dimensionless Henry’s constant
$H_{i}$	Hydraulic dissipate energy (J kg^−1^), i=carbon fiber, empty
$I_{E}$	Absorption flux improvement
$I_{P}$	Power consumption relative index
$k_{a}$	Mass transfer coefficient in the CO_2_/N_2_ gas feed stream (m s^−1^)
$k_{b}$	Mass transfer coefficient in the MEA absorbent feed stream (m s^−1^)
$K_{ex}$	Equilibrium constant
$K_{ex}^{\prime }$	Reduced equilibrium constant
$K_{m}$	Overall mass transfer coefficient of membrane (m s^−1^)
${lw}_{f,j}$	Friction loss (J kg^−1^), $j=CO_{2}/N_{2},\;MEA$
L	Channel length (m)
$M_{w}$	Average molecular weight of CO_2_ and N_2_ gas mixture $({kg\;mol}^{-1}$)
$N_{exp}$	Number of experimental measurements
$N_{1}$	Number of carbon fiber filaments
$P_{1}$	Saturation vapor pressure in the gas feed flow side (Pa)
$P_{2}$	Saturation vapor pressure in the liquid absorbent flow side (Pa)
$Q_{a}$	Volumetric flow rate of the gas feed stream ($m^{3}\;s^{-1}$)
$Q_{b}$	Volumetric flow rate of the MEA absorbent stream ($m^{3}\;s^{-1}$)
R	Gas constant (8.314 $J\;{mol}^{-1}\;K^{-1}$)
Re	Reynolds number
Sc	Dimensionless Schmidt number
${Sh}^{c}$	Enhanced dimensionless Sherwood number
${Sh}_{lam}$	Dimensionless Sherwood number for laminar flow
W	Channel width (m)
$W_{1}$	Average carbon fiber width (m)
ω	Absorption flux ($mol\;m^{-2}\;s^{-1}$)
${\left|Y_{m}\right|}_{ln}$	Natural log mean CO_2_ mole fraction in the membrane
z	Axial coordinate along the flow direction (m)
Greek letters	
$\alpha^{c}$	Mass transfer enhancement factor
β	Aspect ratio of the channel
$\delta_{m}$	Thickness of membrane (µm)
ε	Membrane porosity
$\bar{v}\;$	Average velocity ($m\;s^{-1}$)
$\rho_{j}$	Density ($kg\;m^{-3}$), $j={CO}_{2},MEA$
$\gamma_{m}$	Concentration polarization coefficients
Subscripts	
1	Membrane surface in CO_2_/N_2_ gas feed side
2(l)	Liquid phase on membrane surface on MEA feed stream
2(g)	Gas phase on membrane surface on MEA feed side
*a*	The CO_2_/N_2_ gas feed stream
*b*	The MEA absorbent feed stream
*cor*	Correlated results
*carbon fiber*	Inserting carbon fibers as promoters
*empty*	Empty channel
*exp*	Experimental results
g	Gas feed stream
*in*	At the inlet
l	MEA absorbent feed stream
*out*	At the outlet
*theo*	Theoretical predictions
Superscripts	
*as*	Ascending carbon fiber width operations
*con*	Uniform carbon fiber width operations
*des*	Descending carbon fiber width operations
