# Supplementary material for: Effects of Hydraulic Diameters on CO2 Absorption in Flat-Plate Membrane Contactors with Inserted S-Ribbed Carbon Fiber Turbulence Promoters
Source: Membranes (Basel). 2026 Apr 30;16(5):162. doi: 10.3390/membranes16050162 (PMC13208148; doi:10.3390/membranes16050162)
Supplement: Supplementary file 1 [file membranes-16-00162-s001.zip › membranes-4130070-supplementary.pdf]

Supplementary Materials:

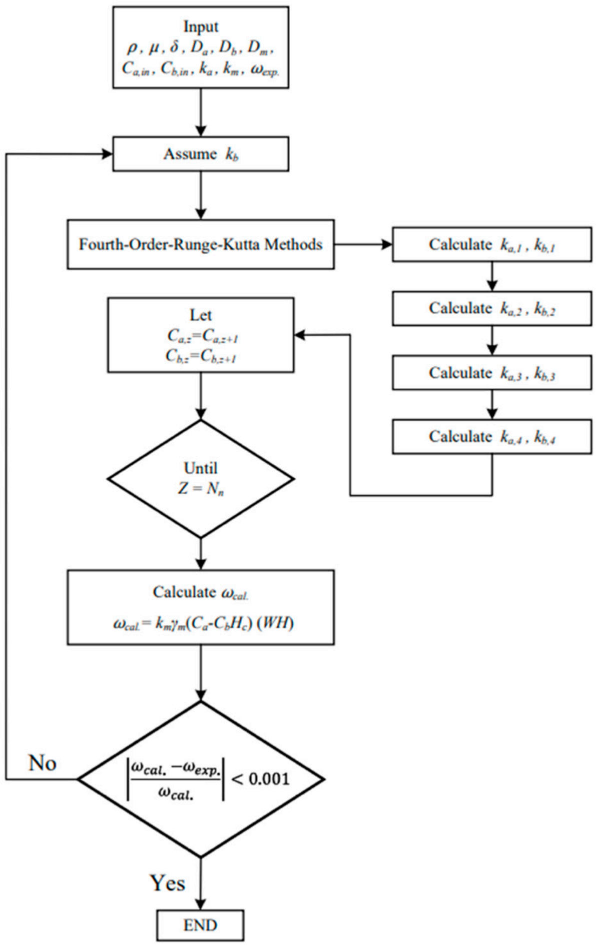

Figure S1. Flow chart for determining concentrations in both CO_2/N_2 and MEA feed streams

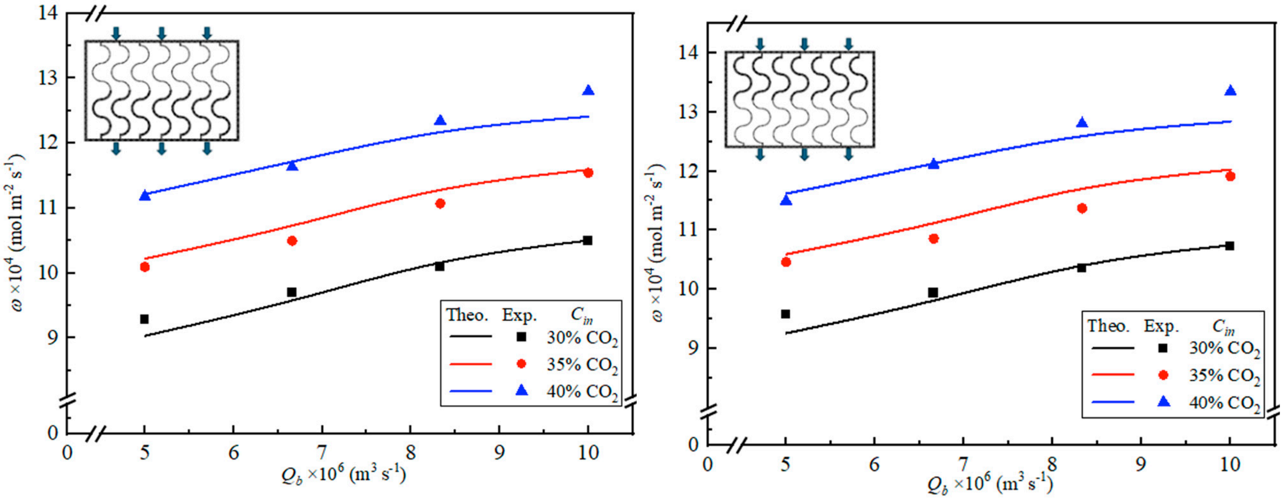

(a) Ascending 3–5 mm carbon-fiber widths

(b) Descending 5–3 mm carbon-fiber widths

Figure S2. Effects of MEA flow rate and inlet CO_2 feed concentration on CO_2 absorption flux under ascending and descending carbon-fiber-width configurations.

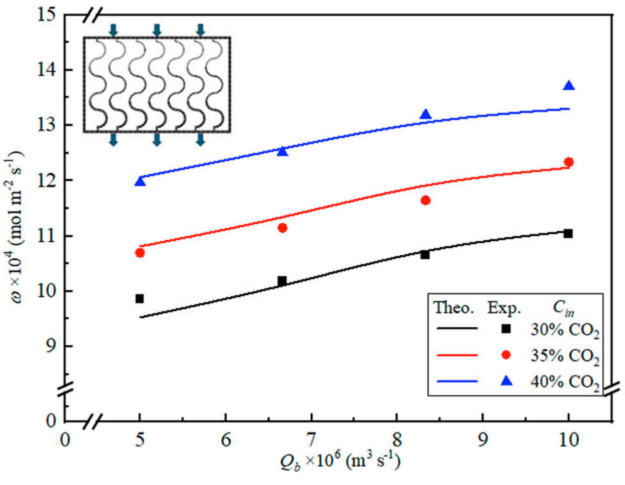

(a) Ascending 3–4–5 mm carbon-fiber widths

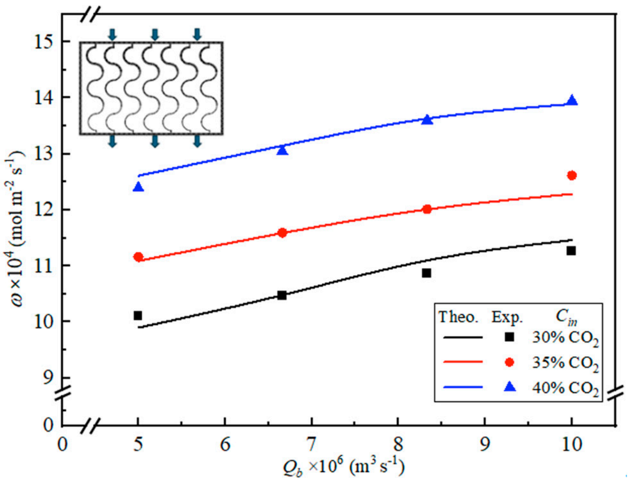

(b) Descending 5–4–3 mm carbon-fiber widths

Figure S3. Effects of MEA flow rate and inlet CO_2 feed concentration on CO_2 absorption flux under ascending and descending carbon-fiber-width configurations.

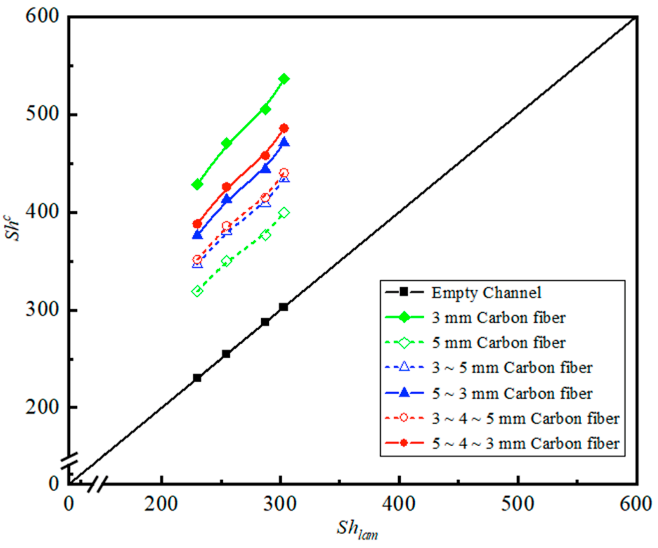

Figure S4. Comparison of correlated and experimental Sherwood numbers for empty channel and S-ribbed carbon-fiber channel under both descending and ascending operations ($C_{in} = 35\%$).

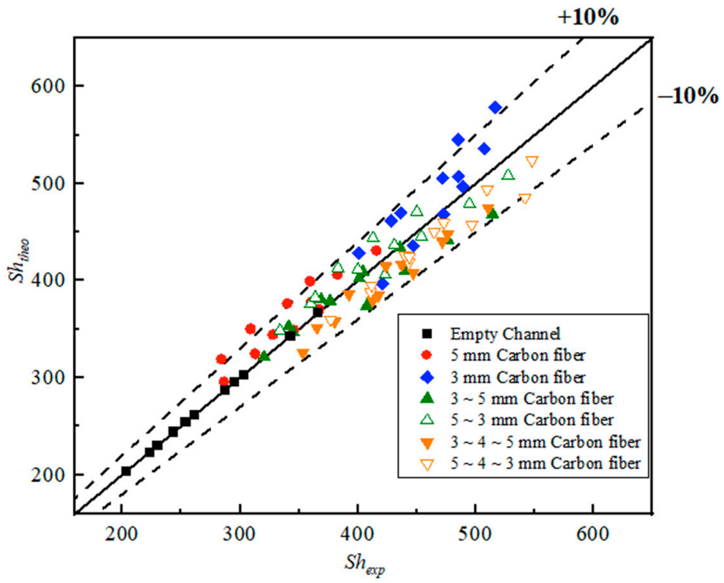

Figure S5. Comparisons between the correlated and experimental Sherwood numbers.
